# Reconstitution and NMR Characterization of the Ion-Channel Accessory Subunit Barttin in Detergents and Lipid-Bilayer Nanodiscs

**DOI:** 10.3389/fmolb.2019.00013

**Published:** 2019-03-14

**Authors:** Thibault Viennet, Stefanie Bungert-Plümke, Shantha Elter, Aldino Viegas, Christoph Fahlke, Manuel Etzkorn

**Affiliations:** ^1^Institute of Physical Biology, Heinrich-Heine-University Düsseldorf, Düsseldorf, Germany; ^2^Institute of Complex Systems 6, Forschungszentrum Jülich, Wilhelm-Johnen-Strasse, Jülich, Germany; ^3^Institute of Complex Systems 4, Forschungszentrum Jülich, Wilhelm-Johnen-Strasse, Jülich, Germany

**Keywords:** barttin, ion channel, lipid bilayer nanodisc, detergent micelle, nuclear magnetic resonance

## Abstract

Barttin is an accessory subunit of ClC-K chloride channels expressed in the kidney and the inner ear. Main functions of ClC-K/barttin channels are the generation of the cortico-medullary osmotic gradients in the kidney and the endocochlear potential in the inner ear. Mutations in the gene encoding barttin, *BSND*, result in impaired urinary concentration and sensory deafness. Barttin is predicted to be a two helical integral membrane protein that directly interacts with its ion channel in the membrane bilayer where it stabilizes the channel complex, promotes its incorporation into the surface membrane and leads to channel activation. It therefore is an attractive target to address fundamental questions of intermolecular communication within the membrane. However, so far inherent challenges in protein expression and stabilization prevented comprehensive *in vitro* studies and structural characterization. Here we demonstrate that cell-free expression enables production of sufficient quantities of an isotope-labeled barttin variant (I72X Barttin, capable to promote surface membrane insertion and channel activation) for NMR-based structural studies. Additionally, we established purification protocols as well as reconstitution strategies in detergent micelles and phospholipid bilayer nanodiscs. Stability, folding, and NMR data quality are reported as well as a suitable assignment strategy, paving the way to its structural characterization.

## Introduction

ClC-K channels form a subgroup of chloride channels within the ClC family of chloride channels and chloride/proton antiporters. They are expressed in the kidney and in the inner ear, and are essential for NaCl re-absorption in the loop of Henle and for potassium secretion by the stria vascularis (Fahlke and Fischer, 2010; Stolting et al., 2014). Barttin is the accessory β-subunit of ClC-K channels. It is the gene product of *BSND*, the disease gene of a rare human disease, called Bartter syndrome IV. Patients suffer from a severe impairment of urinary concentration ability as well as sensorineural deafness (Landau et al., 1995; Rickheit et al., 2008; Riazuddin et al., 2009; Tan et al., 2017). Barttin has been shown to be necessary for the function of both chloride channels ClC-Ka and ClC-Kb (Estevez et al., 2001) present, respectively, in inner ears and in kidneys. Barttin modifies protein stability, promotes its insertion into the plasma membrane, and turns the channel into a conductive state (Hayama et al., 2003; Scholl et al., 2006).

The predicted transmembrane topology of barttin consists of a short cytoplasmic N-terminus of eight amino acids, two transmembrane helices encompassing the amino acids between 9 and 54 and a large cytoplasmic C-terminus. Whereas, the transmembrane core of barttin including the short cytoplasmic N-terminus is sufficient for fulfilling the effects on channel stability and intracellular trafficking, amino acids in the C-terminus are required for normal function of ClC-K/barttin channels (Scholl et al., 2006; Fischer et al., 2010; Steinke et al., 2015; Wojciechowski et al., 2018). Co-immunoprecipitation studies and confocal microscopy indicate binding of barttin to the B- and/or J-helix at the outer surface of the pore-forming subunit of ClC-K channel (Tajima et al., 2007). Despite this model and features, the structural characteristics of barttin remain elusive, pointing to the need for proper investigation of the protein, aiming to better understand the pathophysiology, the effect of *BSND* mutations (Steinke et al., 2015; Wojciechowski et al., 2018), and hoping to discover targets for drug development (Tajima et al., 2007).

While detergent micelles are most commonly used for structural studies of membrane proteins, they exhibit severe destabilization (Seddon et al., 2004) and denaturation effects (Zhou and Cross, 2013). Phospholipid bilayer nanodiscs (NDs) have proven to be a useful alternative membrane mimetic (Bayburt and Sligar, 2010; Nasr et al., 2017; Viennet et al., 2018). They have the advantage of allowing the use of numerous types of lipids, and have the power to mimic key properties of membranes thus providing potentially more native environments to membrane proteins. The nanodisc system has been successfully applied in numerous NMR-based studies (Viegas et al., 2016a), including dynamic and structural investigations of β-barrel proteins (Hagn et al., 2013; Fox et al., 2014) as well as the tumor necrosis factor receptor p75NTR (Mineev et al., 2015) which contains a single transmembrane domain or to the seven-transmembrane helices protein bacteriorhodopsin (Etzkorn et al., 2013). Although NMR-optimized constructs have been developed (Hagn et al., 2013), NDs are still a challenging environment for high resolution NMR studies due to their overall large size. As such, pursuing a combined approach using both detergent micelles and nanodiscs in order to exploit the good NMR properties of micelles and the presumably more native environment of the nanodiscs can be a valid approach. This was done in the case of Opa_60_, where restraints derived from NMR in both dodecylphosphocholine (DPC) and 1,2-dimyristoyl-*sn*-glycero-3-phosphocholine (DMPC) bilayer NDs were used (Fox et al., 2014).

Here we report on expression, purification and reconstitution properties of barttin, which so far eluded a comprehensive *in vitro* characterization. The usage of an *E. coli* based cell-free protein expression system enabled production of larger quantities of barttin that we used for reconstitution procedures both in detergent micelles and in phospholipid bilayer nanodiscs. For the latter the influence of lipid and detergent types as well as detergent removal procedures on the quality of resulting nanodiscs was systematically evaluated. Using the optimized protocols, barttin could be successfully solubilized in detergent micelles as well as refolded into nanodiscs in sufficient quantities for subsequent structural and biophysical characterization. As expected NMR spectral quality is largely improved in detergent micelles, however our data show that barttin is considerably less stable in the used micelle system as compared to nanodiscs. While barttin adopts a secondary structure in detergent micelles consistent with its predicted behavior, the NMR spectra in micelles and nanodiscs differ more than expected for the changing environments, indicating that the two environments do not necessarily stabilize the same barttin structure.

## Materials and Methods

### Cell-Free Expression

A barttin construct containing the N-terminal 72 residues of barttin preceded by a His-tag (His-I72X) was cloned into a pET21a vector and plasmid DNA was produced. Barttin was expressed in an *E. coli*-based cell-free expression system following established protocols (Schwarz et al., 2007; Klammt et al., 2012). For NMR samples expression was carried out using deuterated buffers (>90% D_2_O) and either triple (^2^H, ^13^C, ^15^N) labeled Algal extract or custom-made mix with selected amino acids with different isotope labeling suitable for combinatorial assignments was used. Since the Algal isotope mixture lacks the four amino acids: Cys, Trp, Gln, and Asn, the missing amino acids were added in natural abundance at final concentrations of 1 mM. The combinatorial test sample contained the following labeling pattern: ^15^N-Val, ^15^N-Cys, ^15^N-Leu, ^15^N-Ser, ^13^C'-Met, u-(^13^C;^15^N)-Val and all other amino-acid types at natural abundance. Dialysis mode reactions were carried out at 28°C with or without the presence of nanodiscs. After 12–16 h, the reaction mix was centrifuged for 10 min at 12,000·*g*. The pellet was washed once with 5 to 10 volumes of buffer containing 50 mM NaH_2_PO_4_ pH 8.0, 300 mM NaCl (buffer A, all reagents from Sigma Aldrich if not stated otherwise) and Complete protease inhibitors (Roche). The resulting pellet was stored at −20°C until further use.

### Purification and Detergent Solubilization

For detergent solubilization tests, pellets were directly solubilized in 50 mM NaH_2_PO_4_ pH 8.0, 150 mM NaCl supplemented with either 20 mM sodium dodecylsulfate (SDS), 100 mM decylphosphocholine (FOS-10, Cube Biotech) 100 mM dodecylphosphocholine (FOS-12 or DPC, Cube Biotech), 100 mM *N,N*-dimethyldodecylamine *N*-oxide (LDAO, Cube Biotech), 42 mM lyso-myristoylphosphatidylglycerol (LMPG, Avanti polar lipids), 250 mM n-decyl-β-*D*-maltoside (DM, Anatrace) or 196 mM n-dodecyl-β-*D*-maltoside (DDM, Cube Biotech) in a thermomixer (Eppendorf) at 37°C, 800 rpm for 2 h, without further purification.

For small-scale NMR samples and combinatorial-labeled samples, pellets were directly solubilized with NMR buffer (20 mM NaPi pH 7.0, 100 mM NaCl, 2 mM TCEP, 0.2% (v/v) NaN_3_ and 10% (v/v) D_2_O) supplemented with either 100 mM DPC, 42 mM LMPG or 100 mM LDAO in a thermomixer (Eppendorf) at 37°C, 800 rpm for 2 h without further purification.

For large-scale triple-labeled (^2^H,^13^C,^15^N) samples, pellet was solubilized at room temperature for 30 min with 10 volumes of buffer A supplemented with 2 mM DTT, 50 mM LDAO, and Complete protease inhibitors, then centrifuged at room temperature at 16,000·*g* for 30 min. The supernatant was incubated with previously washed Ni-NTA agarose chemical beads (Macherey-Nagel) at room temperature for 1 h. The slurry was transferred to a column, washed with 10 column volumes of buffer A supplemented with 2 mM DTT and 10 mM LDAO. Barttin was eluted using buffer A supplemented with 2 mM DTT, 10 mM LDAO and 300 mM imidazole, fractions containing Barttin were pooled and applied to a desalting column equilibrated with NMR buffer supplemented with 10 mM LDAO. Finally, the eluate was concentrated in a 10 kDa cutoff Vivaspin concentrator.

### Reconstitution in Lipid Bilayer Nanodiscs

#### Membrane Scaffold Protein Preparation

*E. coli* BL21 (DE3) were transformed with the MSP1D1 or MSP1D1Δ5 plasmid DNA in a pET28a vector as reported in Ritchie et al. (2009); Hagn et al. (2013). In short, cells were grown in LB medium. Protein was resuspended with 6M Gdn-HCl and purified by IMAC (without denaturating agent). The elution fractions were pooled and dialyzed in order to remove imidazole. N-terminal His-tag was cleaved using TEV protease incubated overnight at 4°C. ΔHis-MSP1D1 or ΔHis-MSP1D1Δ5 was separated from MSP1D1 or MSP1D1Δ5 by IMAC.

#### Barttin Purification in SDS

Barttin was purified from washed CFE pellets to SDS following similar procedure as in LDAO. In short, pellets were solubilized in buffer containing 20 mM SDS, supernatant was diluted to 10 mM SDS previous to binding to Ni-NTA agarose beads. Either the slurry or the pooled elution fractions were used for nanodiscs assembly.

#### Nanodiscs Assembly

Barttin in SDS, ΔHis MSP1D1 or MSP1D1Δ5 (6-fold molar excess over barttin) and lipids (450-fold molar excess over barttin for MSP1D1 or 270-fold for MSP1D1Δ5) solubilized in 60 mM Na-cholate were mixed together in 20 mM Tris-HCl pH 7.5, 100 mM NaCl, 2 mM DTT, 10 mM SDS. Different lipids (all from Avanti polar lipids) were used including 1,2-dimyristoyl-*sn*-glycero-3-phosphocholine (DMPC), a mixture of 1-palmitoyl-2-oleoyl-*sn*-glycero-3-phospho-(1′-*rac*-glycerol (POPG) and 1-palmitoyl-2-oleoyl-*sn*-glycero-3-phosphocholine (POPC) in the ratio 1:4 and a mixture of 1,2-distearoyl-*sn*-glycero-3-phospho-L-serine (DSPS), 1,2-dipalmitoyl-*sn*-glycero-3-phosphocholine (DPPC), 1,2-distearoyl-*sn*-glycero-3-phosphocholine (DSPC), and 1,2-diarachidoyl-*sn*-glycero-3-phosphocholine (DAPC) in ratio 1:3:3:3 (Mitchell et al., 2007; Kim et al., 2013). Lipids in chloroform were tried under nitrogen flow and stored under vacuum before usage.

Detergent removal procedure was empirically optimized, different approaches including usage of Biobeads SM-2 (Biorad) on-column or in the purified product, dialysis through a 10 kDa cutoff membrane (Thermo Scientific), on-column fast and stepwise wash of the detergent were tested. For more information see Results section.

For all off-column procedures an additional IMAC purification was done in order to separate nanodiscs containing Barttin from those which did not. Finally, Barttin-containing NDs were purified by SEC on a HiLoad 16/600 Superdex 200 pg (for large scale) or Superdex 10/300 gl (for small scale) column (GE Healthcare) equilibrated with 20 mM sodium phosphate pH 7.0, 100 mM NaCl, 2 mM DTT using a ÄKTA pure device running at 1 ml/min. The main peak corresponding to expected NDs was pooled and concentrated using a Vivaspin centrifugal device of 10 kDa MWCO. For NMR samples, final concentrations of 2 mM TCEP, 10% v/v D2O and 0.01% v/v NaN_3_ were added before measurements.

For co-translational incorporation of Barttin into NDs, empty nanodiscs assembled with DMPC according to established protocols (Denisov et al., 2004; Ritchie et al., 2009) were used in the cell-free system's reaction mix at a concentration of 58 μM.

### SDS-PAGE

Proteins were analyzed using denaturing, non-continuous tricine-sodium dodecyl sulfate-polyacrylamide gel electrophoresis according to published methods (Schägger and von Jagow, 1987). The gel consists of three parts, a 17% acrylamide separation gel, a 10% intermediate gel and a 5% stacking gel. To separate the proteins in an electric field, the following anode buffer (200 mM Tris-HCl pH 8.9) and cathode buffer (200 mM Tris-HCl, 100 mM Tricine and 0.1% SDS) were used. The PageRuler Plus prestained protein ladder (ThermoFisher) was used as protein standard. Proteins were visualized after Coomassie blue staining.

### Circular Dichroism

A sample of 10 μM Barttin in LDAO micelles was investigated using circular dichroism (Jasco J-715) in 20 mM NaPi pH 6.8, 100 mM NaCl, 2 mM TCEP and at room temperature. Measurement from 260 to 200 nm was done at a constant bandwidth of 1 nm, with a data pitch of 0.2 nm and repeated 10 times for averaging. Data was converted to mean-residue molar ellipticity and convoluted using the K2d protocol.

### NMR Data Acquisition

NMR experiments were performed on Bruker Avance III HD^+^ spectrometers operating either at 600 or 700 MHz, both equipped with a triple resonance TCI (^1^H, ^13^C, ^15^N) cryoprobe. Data was collected at 35°C in 20 mM NaPi pH 6.8, 100 mM NaCl, 2 mM TCEP, 0.2% (v/v) NaN_3_, and 10% (v/v) D_2_O. For experiments using LDAO micelles, the detergent concentration after concentration was estimated to roughly 100 mM. All NMR experiments contained transverse relaxation optimized spectroscopy (TROSY) components (Pervushin et al., 1997; Salzmann et al., 1999). The 2D ^13^CO- and ^13^Cα-filtered TROSY-HSQC pulse sequences were designed from their respective 3D experiments. All NMR spectra were processed with TOPSPIN 3.2 (Bruker) and analyzed with CARA (Keller, 2004) and CCPN (Vranken et al., 2005).

## Results and Discussion

### Barttin Can be Expressed in a Cell-Free Setup and Incorporated Into Nanodiscs

In general, structural biology aspires to obtain data in a native environment of the target system in order to ensure relevant folding and necessary cofactors for function, without introducing bias due to *in vitro* handling procedures. While some techniques theoretically have the power to do so, such as in-cell NMR (Theillet et al., 2016) or dynamic nuclear polarization NMR (Viennet et al., 2016), they often face limitations in sensitivity and/or resolution, which is particularly true for membrane systems. For membrane proteins, the presence of a phospholipid bilayer with similar chemical composition and physical properties as the native membrane is evidently one of the most important environmental factors. When pursuing *in vitro* investigations, nanodiscs, as compared to other membrane mimetics such as detergent micelles, bicelles or, amphipols, exhibit several key advantages (Bayburt et al., 2002): (i) they provide homogenous lipid-bilayer particles, (ii) both sides of the bilayer are accessible in solution, (iii) they have high stability and low exchange rates of lipid and protein molecules between different NDs, (iv) the presence of the scaffold protein may mimic a crowded environment as often found in natural membranes, and (v) a large range of lipids (charge, length, unsaturations, non-phospholipids, etc.) can be incorporated allowing mimicking of properties of various types and states of membranes (Viegas et al., 2016a).

Structural studies of membrane proteins are often impeded by limitations in protein expression due to cytotoxic effects of the target protein in its heterologous overexpression systems. Unfortunately, barttin belongs to those membrane proteins that are difficult to produce in large quantities in conventional expression systems (unpublished observations). Therefore, so far, all published barttin data resulted from in cell experiments where protein amount is not critical (Scholl et al., 2006; Fahlke and Fischer, 2010; Fischer et al., 2010). Cell-free expression (CFE) offers a valuable alternative, which also has the advantage of supporting custom labeling strategies thanks to the direct use of amino acids and the very low level of metabolic scrambling (Katzen et al., 2009). We tested expression of barttin in an *E. coli*-based cell-free expression system. Following established protocols (Schwarz et al., 2007) we could obtain sufficient quantities of protein for subsequent characterization.

We initially carried out CFE in the absence of membrane mimetics. This strategy requires reconstitution of the resulting protein pellet into a suitable membrane mimetic. While canonical protocols for detergent reconstitution or incorporation into nanodiscs exist, they need to be empirically optimized for each target protein. For the latter factors such as the target protein to scaffold protein to lipid ratio, the detergent type and removal procedure may strongly differ for different target proteins (Viegas et al., 2016a).

To test barttin incorporation into nanodiscs we started with the following setup: SDS was used to solubilize barttin CFE pellets, MSP1D1 (D1) was used as scaffold protein, DMPC lipids were solubilized in sodium cholate, and adsorbent polystyrene beads were used to remove detergents. Although empty NDs assembly was detected, no detectable levels of barttin were incorporated with this setup (data not shown, see Table 1). Subsequently we changed the detergent in which lipids are solubilized to SDS, and the lipid mixture to DMPG:DMPC 1:4 (20% negatively charged head groups). While previously shown to largely improve quality of the data (Hagn et al., 2013), it did not lead to detectable improvements for barttin. The most probable explanation is that barttin, a rather hydrophobic and low molecular weight (10.1 kDa) protein, could adsorb to the polystyrene beads.

**Table 1:** Summary of tested conditions for barttin reconstitution in NDs.

**Scaffold**	**Detergent for barttin**	**Detergent for lipids**	**Lipids**	**Detergent removal**	**Yield (%)**
D1	SDS	Na-cholate	DMPC	Biobeads	x
D1	SDS	SDS	DMPC	Biobeads	x
D1	SDS	Na-cholate	DMPG:DMPC	Biobeads	x
D1	SDS	SDS	DMPG:DMPC	Biobeads	x
D1	DPC	Na-cholate	DMPC	Dialysis	x
D1	DPC	Na-cholate	DMPG:DMPC	Dialysis	x
D1	SDS	Na-cholate	DMPC	Dialysis	< 1%
D1	SDS	Na-cholate	DMPG:DMPC	Dialysis	< 1%
D1	SDS	Na-cholate	DMPC	On-column step dilution	x
D1	SDS	Na-cholate	DMPC	On-column fast dilution	4.7
D1	SDS	Na-cholate	DMPC	On-column biobeads	8.2
D1	SDS	Na-cholate	DMPC	Dialysis	5.8
Δ5	SDS	Na-cholate	DMPC	On-column biobeads	14
Δ5	SDS	Na-cholate	“Native” mix	On-column biobeads	9
Δ5	SDS	Na-cholate	DMPC	Dialysis	19.3
Δ5	SDS	Na-cholate	“Native” mix	Dialysis	3.7
Δ5	SDS	Na-cholate	DMPC	Dialysis / Up-scale	8.2
Δ5	SDS	Na-cholate	“Native” mix	On-column biobeads / Up-scale	6.2
Δ5	None	None	DMPC	Co-translationally in CFE	12.8

*Type of scaffold protein, detergents, lipids as well as detergent removal procedure are indicated together with the respective yields (x = not incorporated into NDs)*.

**Figure 1:**
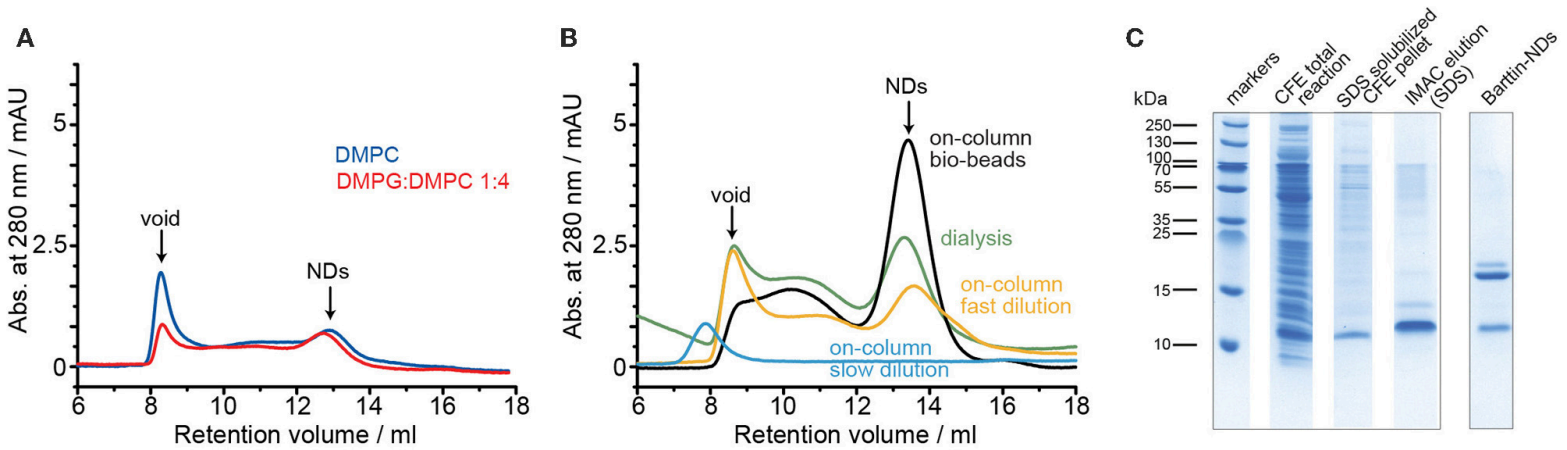
Optimization of barttin reconstitution into D1 NDs. **(A)** SEC profiles of barttin NDs (MSP1D1) assembled with DMPC (blue) and DMPG:DMPC 1:4 (red) upon sodium cholate dialysis. **(B)** SEC chromatograms of barttin NDs (MSP1D1) assembled with DMPC upon detergent dialysis (green), on-column biobeads adsorption (black), on-column fast dilution (yellow), and on-column step dilution (blue). **(C)** Coomassie blue-stained SDS-PAGE of CFE expressed barttin (total reaction), solubilized CFE pellet, SDS- purified barttin and purified (IMAC, before SEC) barttin NDs (MSP1D1) assembled with DMPC upon on-column biobeads adsorption.

In the next step we therefore used dialysis to remove the detergent for ND assembly. The same conditions concerning lipids and detergents were tested and indeed successful incorporation of barttin in nanodiscs was detected (Figure 1A and Table 1). Unfortunately, the yield, defined as the recovery of barttin from the input to the purified NDs, remained very low (< 1%; see Table 1). Changing detergent for barttin solubilization to DPC micelles, which provided good results in other cases (Hagn et al., 2013; Fox et al., 2014), did not improve barttin incorporation. In addition (and possibly in line) with the low yield, the size exclusion profiles also reveal rather poor homogeneity (Figure 1A).

Having found initial conditions allowing barttin incorporation into nanodiscs, additional parameters were screened in order to improve yields and homogeneity of the preparations. Since the detergent removal step seemed critical, three different approaches were tested using on-column procedures successfully reported before (Katayama et al., 2010). In short, Barttin in SDS was immobilized through its histidine tag to a Ni-NTA agarose column, incubated with MSP and lipids and the detergents were removed either by introduction of adsorbent beads, fast dilution of the slurry, or stepwise washing procedure. The latter did not allow any ND assembly, which we attribute to a too slow detergent removal rate, kinetically favoring lipid aggregation over nanodisc formation as seen from the SEC profile (Figure 1B). The other protocols were successful in producing substantial amounts of barttin-containing NDs, with an overall higher homogeneity and yield for the on-column polystyrene beads adsorption of detergents (Figure 1B and Table 1).

In addition to size homogeneity, another important factor in ND reconstitution is the resulting oligomeric state of the membrane protein, which is difficult to control as well as to measure accurately (Tsukamoto et al., 2010; Viegas et al., 2016a; Peetz et al., 2017). While, due to the lack of larger extra-membranous domains, incorporation of multiple copies of barttin into the NDs not necessarily changes the resulting SEC profile, the amount of barttin per ND can be estimated from the relative band intensities of MSP and barttin on a SDS-PAGE gel. The respective band intensities, after IMAC purification to remove empty NDs, are in line with a single copy of barttin in D1 NDs (Figure 1C).

**Figure 2:**
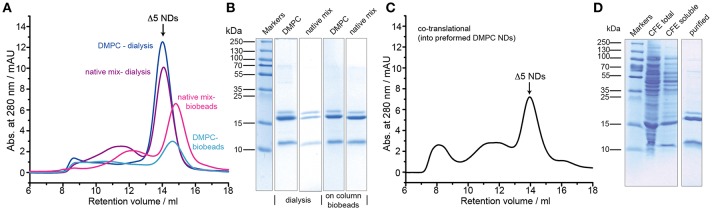
Optimization of barttin incorporation into Δ5 NDs. **(A)** SEC profiles of barttin NDs (MSP1Δ5) assembled with DMPC (blue) and “native-like lipid mix” (purple) upon detergent dialysis, and respective profiles for on-column biobeads adsorption for DMPC (cyan) and lipid mix (magenta). **(B)** Coomassie blue-stained SDS-PAGE gel of the corresponding barttin-NDs samples, purified via IMAC, before SEC. **(C)** SEC profile of barttin NDs formed via co-translational reconstitution of barttin into Δ5 NDs during CFE. **(D)** Coomassie blue stained gel of total CFE reaction in the presence of preformed NDs (MSP1Δ5, DMPC), soluble fraction and IMAC purified barttin NDs.

Smaller nanodiscs, obtained by using shorter MSP variants (Hagn et al., 2013), are known to provide better NMR spectral quality and may provide better control of the oligomeric state for small to medium-sized membrane proteins. We therefore tested the membrane incorporation of barttin also using MSP1D1ΔH5 (Δ5)-based NDs. In this setup we additionally tested the possibility of reconstituting barttin in a more “native-like” phospholipid mixture based on a conserved aliphatic chain lengths distribution and negative charge content (Mitchell et al., 2007; Kim et al., 2013) using a lipid composition of DPPC:DSPC:DAPC:DSPS in a 3:3:3:1 molar ratio. For both standard DMPC and the “native-like lipid mix,” we tested the reconstitution using either dialysis or on-column adsorbent beads removal. In all conditions barttin incorporation was successful and provided sufficient homogeneity of the resulting NDs for NMR studies (Figure 2A). Usage of the “native-like lipid mix,” however, provided lower yields (Figure 2A and Table 1). The oligomeric state of barttin in Δ5 NDs was also estimated to one barttin per ND in all tested conditions (Figure 2B).

Noteworthy the usage of a cell-free expression system also allows to add preformed nanodiscs to the reaction mix to promote co-translational incorporation of the expressed protein directly into the NDs (Rues et al., 2016; Viegas et al., 2016a). We tested this approach using preformed DMPC Δ5 nanodiscs, which yielded fair quality and amount of barttin-containing NDs (Figures 2C,D). However, overall homogeneity and yield was lower as for other reconstitution protocols (see Table 1).

**Figure 3:**
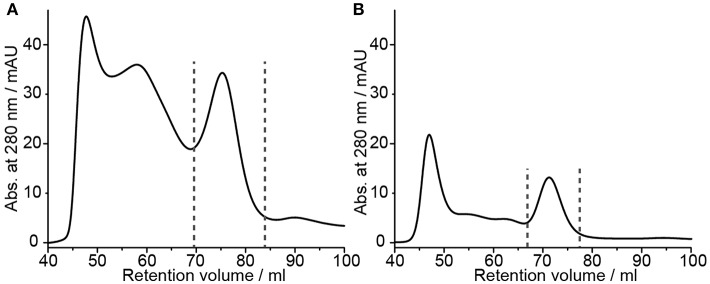
NMR sample preparation of barttin in Δ5 NDs. **(A)** SEC profile of up-scaled barttin-NDs (MSP1Δ5) assembled with “native-like lipid mix” upon on-column biobeads adsorption. **(B)** SEC profile of up-scaled barttin-NDs (MSP1Δ5) assembled with DMPC upon dialysis. Dotted line mark fractions collected in the corresponding NMR samples.

All previous experiments were carried out using a rather low amount of barttin (from two small scale 50 μl cell-free expressions). Since NMR studies require higher amounts of material (in the mg range), we tested up-scaling of the most promising protocols, i.e., “native-like lipid mix” together with on-column detergent adsorption and DMPC together with detergent dialysis. For both setups cell-free pellets from 3 ml CFE reactions were used. In line with general observations in upscaling, in both cases the yields dropped by 30 and 60%, respectively (Figures 3A,B and Table 1). Nevertheless, sufficient amounts of ^15^N-barttin in Δ5 NDs for NMR characterization could be obtained.

Summarizing the screening of the tested conditions, several key points in barttin expression and reconstitution can be concluded, including: (i) CFE offers a convenient approach to prepare high quantities of barttin (ii) cell-free expressed barttin can be easily purified (iii) barttin co-translationally inserts into preformed NDs during cell-free expression (iv) barttin is likely to adsorb into polystyrene beads (v) detergent removal setups and resulting removal rates during barttin-ND self-assembly are very critical parameters, and (vi) various types of phospholipid mixtures lead to successful incorporation of barttin into NDs.

### Barttin in Detergent Micelles

Although providing clear advantages, nanodiscs can still be challenging for NMR-based studies due to their larger size as compared to detergent micelles (~30 kDa for barttin in LDAO micelles as compared to 110 kDa for Barttin in Δ5 NDs). Therefore, in cases where protein structure and function are not corrupted by the detergent, detergent micelles can facilitate NMR-based characterization.

**Figure 4:**
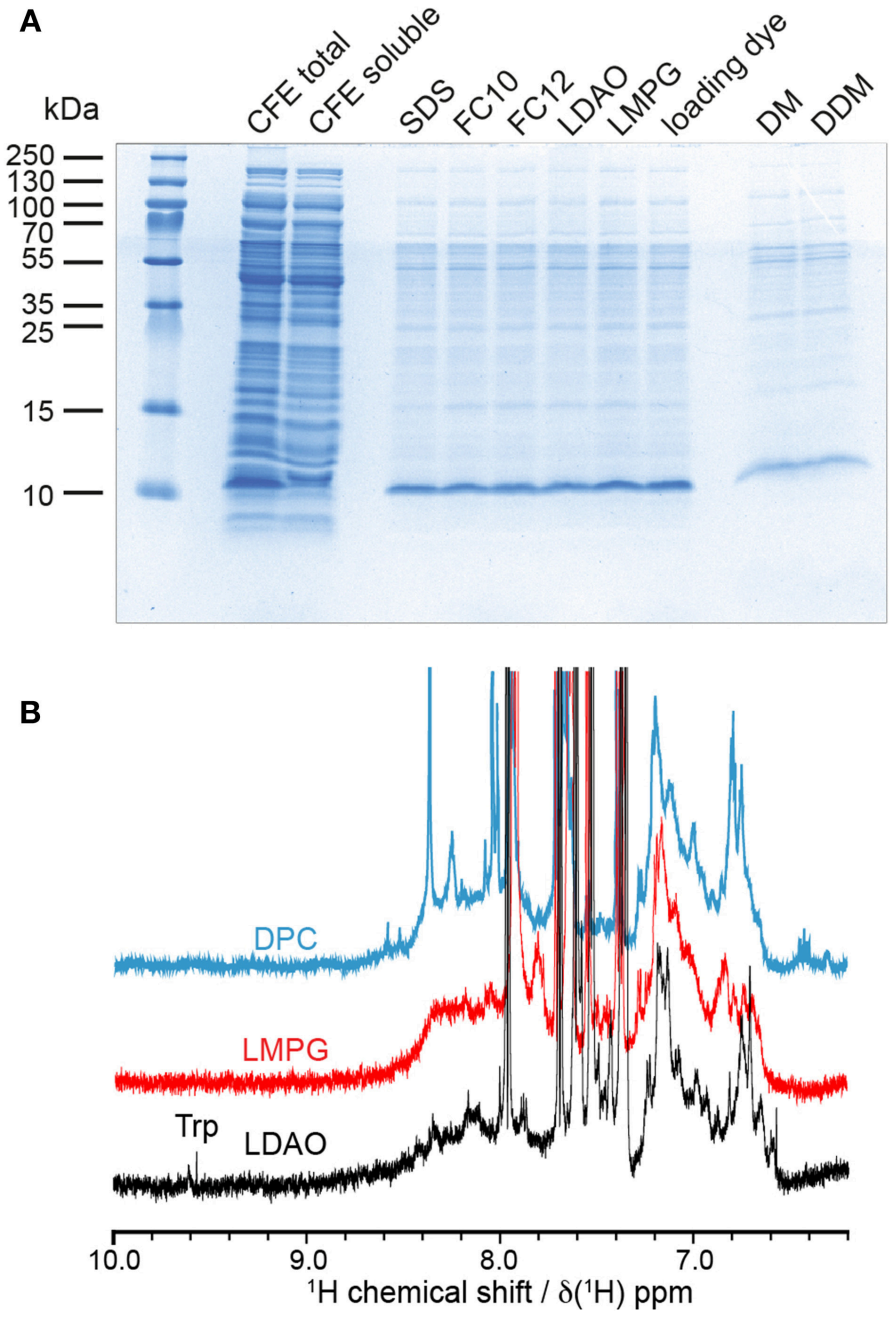
Solubility and NMR properties of barttin in different detergent micelles. **(A)** Coomassie stained SDS-PAGE solubility screen using CFE pellets directly solubilized with indicated detergents (for different detergents only the soluble fraction after centrifugation at 20,000 *g* is loaded into the gel). **(B)** 1D ^1^H NMR screening of resulting most promising barttin containing micelles.

To obtain barttin in detergent micelles, we initially performed detergent screening by directly solubilizing barttin from CFE protein pellets following previous approaches (Klammt et al., 2012). A range of detergents suitable for NMR studies were tested, including FOS-10, DPC, LDAO, LMPG, DM, and DDM (Figure 4A). Interestingly, all tested detergents with the exception of DM and DDM lead to similar solubilization levels as seen from the intensities of the corresponding bands in SDS-PAGE. Three of them were selected for initial NMR screening for their capacity to form small micelles (LDAO, LMPG) or their reported good NMR properties (LDAO, DPC) (Raschle et al., 2009; Hagn et al., 2013). 1D ^1^H NMR spectra of (not isotope labeled) barttin solubilized in the respective micelles without further purification were acquired and compared (Figure 4B). In general, the NMR spectra show an overall similar profile of the amide signals for barttin in the three different detergent micelles. However, the spectrum in LDAO shows several more intense and in particular also better resolved peaks on the flanking region of the amide signals. The latter is an important indicator for NMR-spectral quality (Viegas et al., 2016a). In addition, the appearance of tryptophan side-chain resonances was only observed in LDAO micelles (Figure 4B, Trp-label). We therefore decided to use LDAO micelles for the following steps.

### Barttin in LDAO Micelles

**Figure 5:**
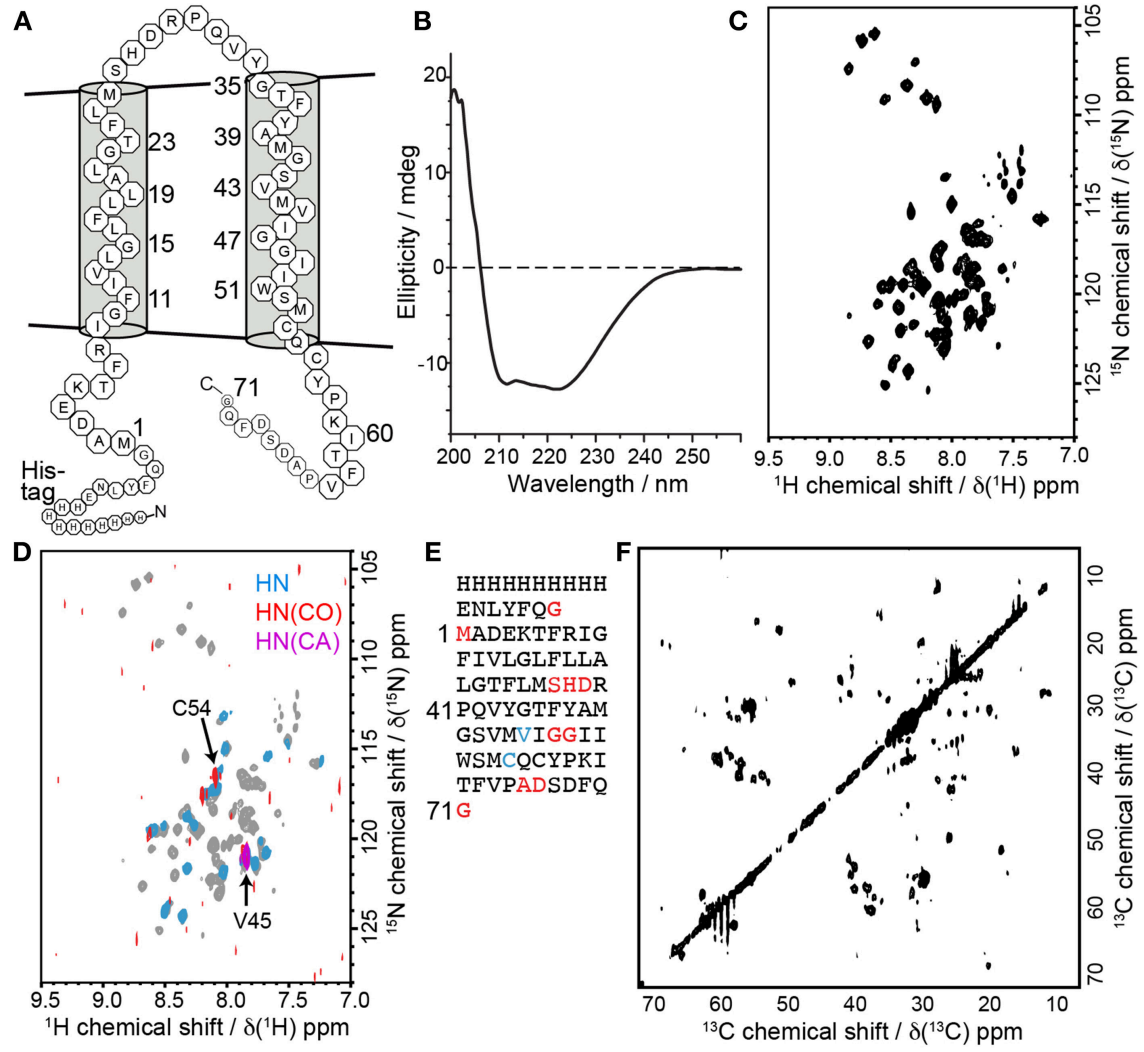
Barttin in LDAO micelles. **(A)** Expected topology model of barttin according to (Wojciechowski et al., 2015). **(B)** Circular dichroism spectrum of barttin in LDAO micelles. **(C)** [^15^N-^1^H]-HSQC spectrum of [U-^2^H-^13^C-^15^N]-labeled barttin in LDAO micelles (Cys, Asn and Trp are unlabeled). **(D)** Overlay of [^15^N-^1^H]-HSQC acquired on [^2^H-^13^C-^15^N]-labeled barttin in LDAO micelles (gray) and on a selectively labeled sample (see methods for labeling pattern, blue). In addition, the [^15^N-^1^H] dimension of a HNCO (red) and of a HNCA (purple) are shown. The labeling pattern directly allows assignments of indicated residues. **(E)** Primary sequence of the used barttin construct highlighting residues with assigned resonance frequencies from conventional 3D experiments (red) and using selective isotope labeling (blue). **(F)** 2D ^13^C-^13^C FLOPSY spectrum acquired using an UTOPIA NMR setup (Viegas et al., 2016b).

In order to further evaluate the characteristics of barttin in LDAO micelles we recorded circular dichroism (CD) data (Figure 5B). As expected for folded barttin the CD spectrum shows a double minimum around 225 and 215 nm, indicative for the presence of α-helical secondary structure. Deconvolution of the CD spectrum (K2d method) indicates a α-helical propensity of 37%. This value is in line with the proposed topology models of barttin (Fahlke and Fischer, 2010), which predict an α-helical content of 44% for the used construct (Figure 5A).

We additionally recorded 2D [^15^N-^1^H]-HSQC NMR data of isotopically labeled barttin in LDAO micelles. The spectrum shows dispersed and mostly resolved peaks (Figure 5C). From the 77 expected peaks about 65 are resolved. Note that cysteine, glutamine, asparagine and tryptophan are not ^15^N-labeled in the used sample. The overall peak positions and dispersion is also in line with the expected features of barttin. To obtain more comprehensive NMR insights into barttin in LDAO micelles we prepared a triple (^2^H, ^13^C, ^15^N) labeled sample of barttin at protein concentration of 250 μM. Note that amide proton back exchange was carried out during the reconstitution process to enable standard ^1^H-detected TROSY-based NMR experiments.

Since ^1^H and ^15^N chemical shift are less reliable secondary structure indicators as compared to ^13^C chemical shifts, we additionally recorded a ^13^C-^13^C correlation spectrum (Figure 5F). Note that the spectrum was obtained for free during the acquisition of a 3D NOESY spectrum using the UTOPIA setup (Viegas et al., 2016b). Evaluation of the amino acids specific ^13^Cα-^13^Cβ peak positions indicates that most residues predominantly occur in random coil and α-helical secondary structure, in line with the CD data and the expected topology model. Taken together these results suggest that barttin adopts a proper secondary structure in LDAO micelles.

Additionally, a set of 3D experiments for resonance assignment was carried out. Unfortunately and reproducibly, NMR spectra of barttin in LDAO micelles change their appearance rather fast (within a few days) indicative of protein degradation and/or aggregation (*vide infra*). The limited stability of barttin in micelles largely reduces the NMR spectral quality due to the alterations of the resonance frequencies of the protein over the time course of the 3D experiments. Consequently, the resulting 3D spectra are difficult to analyze and have lower signal-to-noise ratios than expected, especially when ^13^C-^13^C INEPT transfers are involved. This is often observed in large systems due the unfavorable relaxation properties and may indicate aggregation of barttin micelles. Albeit the spectral challenges, we could obtain resonance assignments for 10 residues (Figure 5E, red).

While the limited sample stability prevents usage of time-intensive 3D NMR experiments, in the case of barttin in LDAO it is still sufficient to obtain 2D spectra, which can be recorded sufficiently fast. Therefore, one possibility to obtain more information in this conditions is to use a combinatorial isotope labeling approach that only requires usage of simple 2D experiments (Parker et al., 2004). The principle is to produce several samples with different types of amino acids that are isotopically labeled with either only ^15^N, only ^13^CO or ^13^C and ^15^N. Comparison of 2D spectra from [^15^N-^1^H]-HSQC, ^13^CO-filtered HSQC, and ^13^Cα-filtered HSQC experiments lead to amino acid type assignment and ultimately to residue-specific assignments. This is made easy by the use of cell-free expression, which allows to introduce single amino acid types with the desired labeling (Lohr et al., 2012, 2015). While a full assignment is not attempted here, we investigated whether this strategy would in general be feasible for barttin in LDAO. We therefore prepared a selectively isotope labeled version of barttin in LDAO micelles and recorded in addition to the 2D ^1^H,^15^N-HSQC spectrum (Figure 5D, blue) also 2D versions of HNCO (Figure 5D, red) and HNCA (Figure 5D, purple) experiments. By using this single sample two additional unambiguous assignment can be made (Figure 5E, blue). We therefore anticipate that usage of a substantial number of differently isotope labeled samples should enable a near complete resonance assignment of the amide resonances. However, since it is not fully clear whether the LDAO embedded state resembles the functional relevant barttin structure (*vide infra*), it is at this point not clear whether this effort would be justified.

### Comparison of Barttin in LDAO Micelles and Nanodiscs

The lack of a reliable functional *in vitro* assay for barttin renders it difficult to judge, whether the protein adopts a relevant state after reconstitution in any membrane mimetic. In this respect it is only possible to compare structural integrity such as secondary structure with the expected behavior as well as to compare NMR-spectra obtained in different membrane mimetics. Under the (in general not necessarily valid) assumption that the NDs are more likely to induce a native-like protein fold, NMR offers the possibility to use spectra obtained in NDs as reference for the protein fold and compare it to the data obtained in detergent micelles (Shenkarev et al., 2009, 2010; Morgado et al., 2015).

One disadvantage of the nanodisc system is that the presence of the scaffold protein impedes light absorption-based experiments such as concentration determination at 280 nm or CD spectroscopy (Viegas et al., 2016a). Thus, the same characterization of barttin secondary structure in NDs is not feasible. However, for NMR measurements signal separation of target and scaffold protein is easily realizable via isotope enrichment of barttin in the CFE setup. We thus carried out an initial NMR characterization of isotope labeled barttin in nanodiscs. Two different ND preparations, i.e., using either 100 % DMPC lipids or using the “native-like lipid mixture” as described above, were used. 2D [^15^N-^1^H]-HSQC spectra of barttin in each of the two ND preparations show a rather small number of resolved peaks indicative of homogenous line broadening of bigger particles and/or heterogenous barttin conformations. Only about 15 out of the expected 77 peaks are visible. The most probable explanation for this is that only signals from residues situated in the protein termini or in the extra-membrane loop appear because of their NMR-favorable dynamic properties. However, without residue-specific assignments, only assumptions can be made.

**Figure 6:**
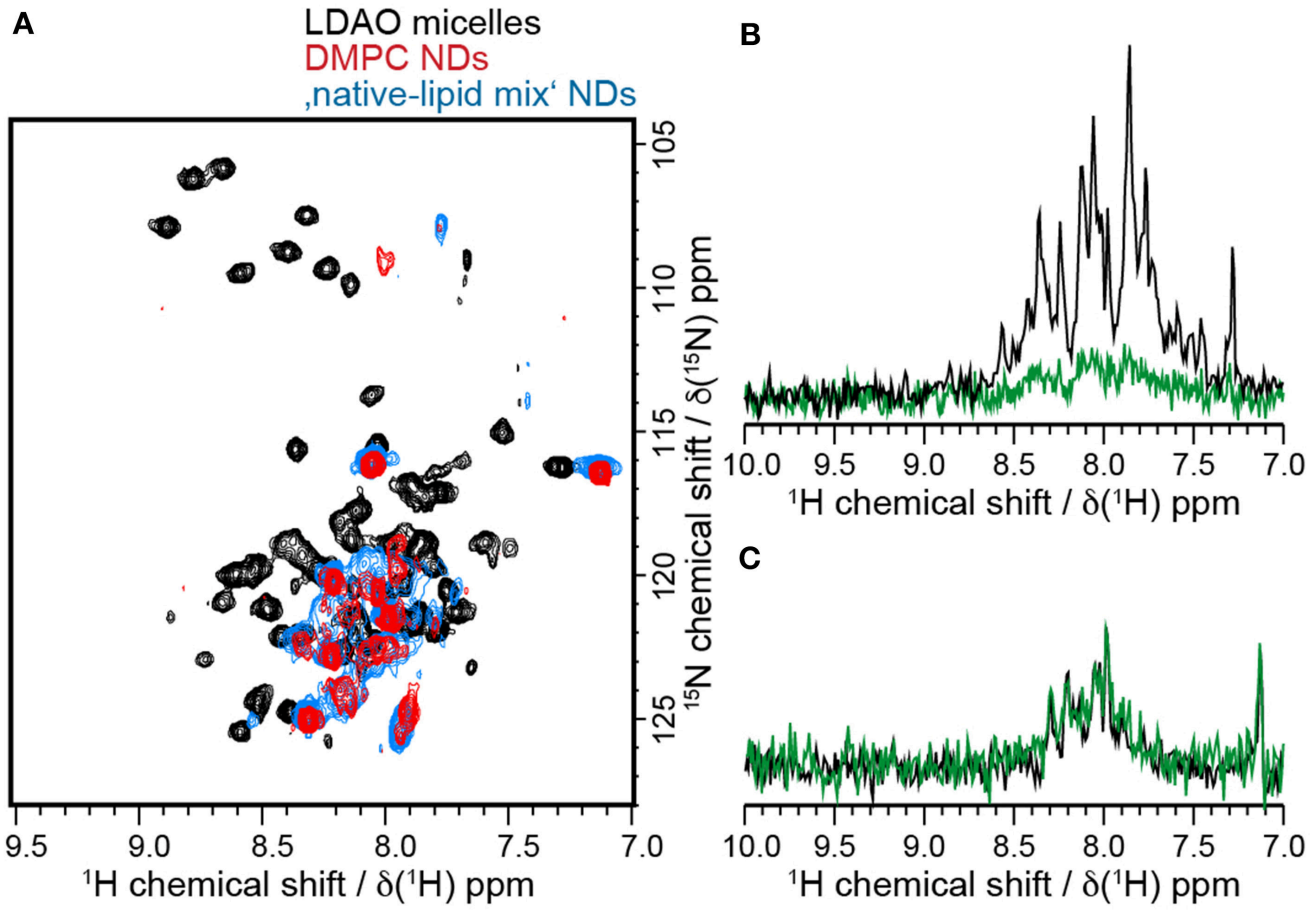
Barttin has different properties in LDAO and NDs. **(A)** [^15^N-^1^H]-HSQC spectra of ^15^N-labeled barttin in LDAO micelles (black), DMPC NDs (red) and “native-like lipid mix” NDs (blue). **(B)** ^15^N-filtered 1D ^1^H NMR spectra of ^2^H-^13^C-^15^N-labeled barttin in LDAO micelles at the beginning of the NMR measurements (black) and after 14 days (green). **(C)** ^15^N-filtered 1D ^1^H NMR spectra of ^15^N-labeled barttin in DMPC NDs at beginning of NMR measurements (black) and after 18 days (green).

When comparing the NMR results obtained in the different NDs to the results in LDAO micelles, it is apparent that the positions of the visible peaks of barttin differ considerably between micelles and nanodiscs (Figure 6A). While the absence of peaks could be explained by the difference in particle sizes, the observed peak shifts indicate that the conformation of the protein is altered in the corresponding region of the protein. Under the assumption that only the extra-membrane residues are observed in the ND samples, this observation would be in line with the denaturation effect of detergents at the micelle-water interface as it was postulated in the case of OmpX (Hagn and Wagner, 2015). Since the conformation and dynamics of this region in general plays an important physiological role, it can at this point not be assumed that barttin in LDAO adopts the same conformation as barttin in nanodiscs.

Interestingly a large overlap between the NMR spectra obtained on barttin in pure DMPC and the “native-like lipid mix” NDs is visible, pointing to rather similar structures. Small changes do arise, which are in line with chemical shift perturbation induced by different membrane mimetic that do not alter protein function as e.g., observed for bacteriorhodopsin in detergent and nanodiscs (Etzkorn et al., 2013).

While NMR data acquired for barttin in LDAO micelles are clearly of much better quality, the stability of the sample is much lower than in NDs. Indeed, as discussed above we could observe gradual degradation of the HSQC signal over time when performing long-time measurements. Over the time course of 14 days a nearly complete disappearance of the signals was observed (Figure 6B). On the contrary barttin in nanodiscs showed no signal decay after 18 days (Figure 6C). This data demonstrates that barttin in the ND-system is considerably more stable as compared to LDAO micelles.

## Conclusion

Our study could identify and overcome several key challenges in the *in vitro* characterization of barttin. We confirm the usefulness of cell-free expression systems for membrane proteins that are challenging to express in bacterial systems (Katzen et al., 2009). Solubilization of resulting CFE protein precipitates using detergent micelles was straightforward for nearly all tested detergents. Nevertheless, considering solubilization yield and NMR spectral quality, our detergent screening for barttin identified LDAO as best micelle environment for our study. CD and NMR data indicate that barttin in LDAO micelles adopts a similar secondary structure as predicted, it is however not possible to conclude that it adopts its native structure.

As an alternative to detergent micelles, we could identify conditions for which cell-free expressed barttin is successfully incorporated into nanodiscs. While the ND incorporation must be empirically optimized for each target, our screening of conditions reveals several features that may be of general relevance for other systems, in particular in respect to the used detergents, the detergent removal procedure and the used lipids. For barttin, usage of SDS for protein solubilization and Na-cholate for lipid solubilization provided best results. While we did not perform a thorough screening of other possible detergents for this step, in our experience and in line with several previous reports (Viegas et al., 2016a), this combination is rather robust and should also be applicable for many other systems. Detergent removal turned out to be the most important aspect of barttin reconstitution into nanodiscs. Our data suggest that barttin is entirely adsorbed to the most commonly used biobeads along with the detergents. This may be similar for other membrane proteins, in particular proteins with only one or two transmembrane helices and small hydrophilic domains. In the case of barttin, dialysis, or immobilization of the protein on a column (IMAC) while using biobeads, provided equally good strategies for detergent removal. Noteworthy, immobilization of the protein on a column during ND assembly can also reduce the number of steps and length of the reconstitution/purification procedure. In respect to the used lipids, we did not observe large effects in ND yield and homogeneity when using the most standard DMPC lipids or a more heterogenous native-like lipid mixture.

Comparison of NMR data of barttin in LDAO micelles or in lipid-bilayer nanodiscs reveals considerable differences reflecting most likely on variations in structure and dynamic of the extra-membrane residues. Interestingly, a comparison of NMR data obtained in nanodiscs formed by the model DMPC lipids are very similar to data obtained on the heterogeneous lipid mixture reflecting the most prominent lipids in barttin's cellular environment. This observation would be in line with the view that predominantly the extra-membranous regions are visible in the NMR spectra of barttin in the used NDs and that, unlike in the case of detergents, these regions are similar for barttin in the model lipid and in the native-like lipid bilayer.

Our data represent the first *in vitro* results of recombinantly expressed and purified barttin and reports on very initial structural results including insights into the effects of different membrane mimetics. While we have invested considerable efforts to overcome several of the major obstacles for the system, our results also suggest that structural characterization of barttin in nanodiscs is still substantially more challenging as expected from its size or as observed for other comparable systems. While barttin in detergent micelles is significantly better accessible via conventional solution NMR approaches, our data show that the protein is not stable in the used LDAO micelles. We demonstrate that combinatorial labeling could overcome this challenge, however our data also show that the barttin structure in micelles likely differs from the one in nanodiscs. Without a reliable *in vitro* functional assay, it can at this point not be decided, which membrane environment is suitable to support barttin's native structure. It is however clear that both tested systems, i.e., detergents and nanodisc, will present challenges in terms of stability or NMR spectral quality, respectively.

Overall several aspects of the presented approach should be transferable to other comparable systems where they may help to optimize sample preparation for structural studies. In respect to barttin we anticipate that our results enable future *in vitro* characterizations of the system, including interaction studies with the ClC-K channels.

## Data Availability

All datasets generated for this study are included in the manuscript and/or the supplementary files.

## Author Contributions

TV, SB-P, SE, and AV conducted the experiments. TV and SB-P analyzed data. TV, SB-P, CF, and ME wrote the manuscript. All authors designed experiments and commented on the manuscript.

### Conflict of Interest Statement

The authors declare that the research was conducted in the absence of any commercial or financial relationships that could be construed as a potential conflict of interest.
